# Historical reconstruction of inaccessibility status in Local Government Areas (LGAs) of Borno and Yobe States, Nigeria, 2010-2020

**DOI:** 10.11604/pamj.supp.2023.45.2.39332

**Published:** 2023-07-14

**Authors:** Joseph Che Forbi, Melton Sundu Musa, Musa Salawu, Jibrin Manu Idris, Ahmed Ibrahim Ba’aba, Jeff Higgins, Audu Idowu Musa, Bamusa Bashir, Aliyu Shettima, Nnamdi Njeakor, Iheanyichukwu Uzoma, Hyeni Mshelia, Gatei wa Nganda, Kabiru Ibrahim Mohammed, Idriss Mohammed Bomoi, Umar Chiroma, Stephanie Diane Kovacs, Oladayo Biya, Ndadilnasiya Endie Waziri, Muyi Aina, Usman Saidu Adamu, Faisal Shuaib, Omotayo Bolu, Richard Franka, Eric Wiesen

**Affiliations:** 1 Polio Elimination Branch, US Centers for Disease Control and Prevention, Atlanta, Georgia, United States of America; 2 African Field Epidemiology Network, Ebonyi State, Nigeria,; 3 Bill and Melinda Gates Foundation, Abuja, Nigeria,; 4 Geospatial Research, Analysis and Services Program, Agency for Toxic Substances and Disease Registry, US Centers for Disease Control and Prevention, Atlanta, Georgia, United States of America,; 5 World Health Organization, Abuja, Nigeria,; 6 Primary Health Care Development Agency, Abuja, Nigeria,; 7 eHealth Africa, Kano, Nigeria,; 8 Solina Center for International Development and Research, Abuja, Nigeria

**Keywords:** Polio, inaccessibility, local government areas, Nigeria

## Abstract

**Introduction:**

ultimately detected in 2016, wild poliovirus (WPV) transmission continued undetected after 2011 in Northeast Nigeria Borno and Yobe States in security-compromised areas, inaccessible due to armed insurgency. Varying inaccessibility prevented children aged <5 years in these areas from polio vaccination interventions and surveillance, while massive population displacements occurred. We examined progress in access over time to provide data supporting a very low probability of undetected WPV circulation within remaining trapped populations after 2016.

**Methods:**

to assess the extent of inaccessibility in security-compromised areas, we obtained empirical historical data in 2020 on a quarterly and annual basis from relevant polio eradication staff for the period 2010-2020. The extent of access to areas for immunization by recall was compared to geospatial data from vaccinator tracking. Population estimates over time in security-compromised areas were extracted from satellite imagery. We compared the historical access data from staff with tracking and population esimates.

**Results:**

access varied during 2010-2020, with inaccessibility peaking during 2014-2016. We observed concurrent patterns between historical recalled data on inaccessibility and contemporaneous satellite imagery on population displacements, which increased confidence in the quality of recalled data.

**Conclusion:**

staff-recalled access was consistent with vaccinator tracking and satellite imagery of population displacments. Despite variability in inaccessibility over time, innovative immunization initiatives were implemented as access allowed and surveillance initiatives were initiated to search for poliovirus transmission. Along with escape and liberation of residents by the military in some geographic areas, these initiatives resulted in a massive reduction in the size of the unvaccinated population remaining resident.

## Introduction

In the eight years before the 2020 certification of the elimination of transmission of indigenous wild poliovirus (WPV) in the World Health Organization (WHO) Region of Africa, Nigeria made substantial inroads toward progress towards enhancing its poliovirus surveillance and improving the quality and reach of polio vaccination [1,2]. A year after the detection of the apparently last WPV in July 2014, the immunization program began preparing to document its country´s polio-free status. However, the last WPV cases in Nigeria were ultimately detected in August and September 2016 among children escaping inaccessible areas in Borno State that were under the control of insurgents [2-4]. Before this discovery, many in the program believed that the inaccessible populations were too small and dispersed to maintain WPV transmission after 2014 [5]. After implementing several innovative immunization and surveillance initiatives, no further WPV cases were found. The WPV-free certification process required a detailed assessment of all the available data by African Regional Certification Commission (ARCC); the polio eradication program in Nigeria conducted extensive investigations, and Nigeria submitted its polio-free certification documentation in August 2019. With additional time and surveillance data, in June 2020, the ARCC accepted the complete documentation for Nigeria and officially certified the WHO Region of Africa as free of all indigenous WPV transmission in August 2020 [6].

The Achilles heel of any disease surveillance and vaccination effort is the inability to conduct activities in inaccessible, security-compromised areas with trapped populations. Areas with violent insecurity prove difficult for public health interventions such as polio vaccination and surveillance, thereby allowing possible undetected, sustained circulation and reemergence of poliovirus or of other vaccine-preventable disease agents [1,7,8]. The identification of four WPV cases in Borno State in 2016 after nearly two years without reported cases refocused attention to insecurity’s role in sustaining and hiding poliovirus transmission; the risk of continued transmission with or without expansion was a major concern for the global polio eradication effort. Generally, there is no or minimal movement in and out of security-compromised areas, limiting participation in polio eradication activities, including routine and supplementary immunization and surveillance activities. There was no systematic data collection on the sizes of relevant trapped population over time. Understanding the dynamic profile of inaccessibility over time was essential in program planning, management and ultimately progressing toward success of the polio eradication initiative.

Since 2009, Borno and Yobe States of Nigeria have been experiencing protracted unconventional armed conflicts with insurgents gaining control of parts of the states, resulting in fluid, fragmented inaccessibility of different areas at different times. Insurgent control of areas expanded in 2012 and peaked in 2014-2016 [9-11]. The main insurgency against the government of Nigeria is perpetrated by the jihadist group, Boko Haram, with the fundamental aim of establishing an Islamic state in the region [12]. The impacts of militant activities of Boko Haram on the polio elimination program included violence against polio workers in the field, the killing of healthcare workers in clinics, and disruption of polio immunization campaigns. As Boko Haram control of parts of the states expanded, trapped populations had reduced or no access to health care and immunization [12].

We sought to reconstruct the extent and dynamics of this geographic inaccessibility of various local government areas (LGAs; equivalent to districts) and some wards (subdistricts) in Borno and Yobe States, Nigeria, during 2010-2020 to provide a framework for understanding poliovirus transmission dynamics in Nigeria. We used traditional sources like notes and personnel files of public health professionals working in those areas and supplemented these by the recollections of those professionals, with the main goal of creating granular maps of inaccessibility over time. This was useful in modelling vaccination and surveillance coverage in those areas to better understand the likelihood of undetected silent WPV circulation continuing as previously seen in South Sudan, Nigeria, Pakistan and Afghanistan due to insecurity and inacessibility [3,13-15].

## Methods

**Figure 1:**
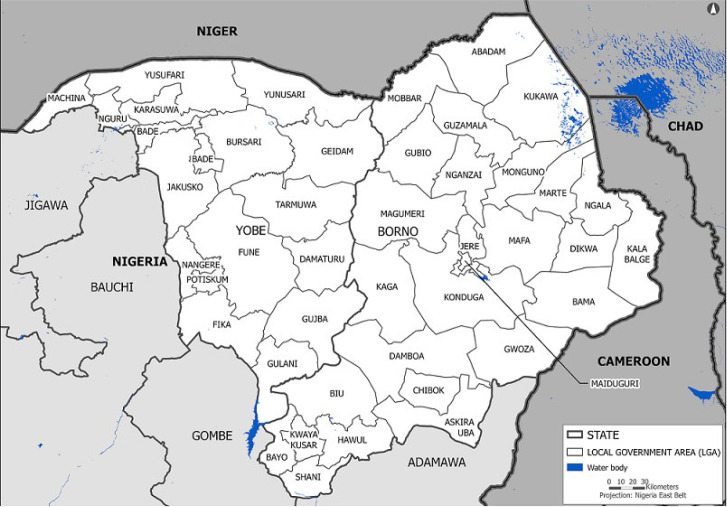
map of Borno and Yobe States, Nigeria showing Local Government Areas (LGAs)

**Setting:** Borno is a state located in North-eastern Nigeria (Figure 1). Maiduguri is the capital and its largest city. The state was formed in 1976 from the split of the North-Eastern State; and until 1991, it included what is now Yobe State. Borno borders the Republic of Niger to the North, Lake Chad (and the Republic of Chad) to the Northeast, and Cameroon to the east; on the south and west, it borders the Nigerian states of Adamawa, Gombe, and Yobe. Borno State consists of 27 LGAs (Abadam, Askira/Uba, Bama, Bayo, Biu, Chibok, Damboa, Dikwa, Gubio, Guzamala, Gwoza, Hawul, Jere, Kaga, Kala/Balge, Konduga, Kukawa, Kwaya Kusar, Mafa, Magumeri, Maiduguri, Marte, Mobbar, Monguno, Ngala, Nganzai and Shani). In 2021, the population of Borno was estimated at close to 6 million inhabitants [16].

Yobe State is also located in North-eastern Nigeria and was carved out of the eastern portion of the prior Borno State in 1991 (Figure 1). The capital of the state is Damaturu; its largest city is Potiskum. The state borders the Diffa and Zinder Regions of Niger Republic to the North. It borders four Nigerian states: Bauchi, Borno, Gombe and Jigawa. Yobe State consists of 17 LGAS (Bade, Bursari, Damaturu, Geidam, Gujba, Gulani, Fika, Fune, Jakusko, Karasuwa, Machina, Nangere, Nguru, Potiskum, Tarmuwa, Yunusari and Yusufari). In 2021, the population of Yobe was estimated at 3 million inhabitants [17]. The two states lie mainly in the dry savanna belt with semi-arid climate prevailing, and as such, conditions are hot and dry for most of the year, except in the southern part of the states, which has more annual rainfall [16,17].

**Study design:** this was a retrospective study.

**Reconstruction of inaccessibility:** data collection was undertaken in early 2021 for all 27 LGAs in Borno and all 17 LGAs in Yobe States, Nigeria, over 2010-2020 (Figure 1). The six LGAs from Yobe were selected because they share geographic boundaries with Borno State (Damaturu, Geidam, Gulani, Gujba, Tarmuwa and Yanusari) and a similar history to armed insurrection by Boko Haram, with occupied areas. Standard questionnaires were designed to elicit qualitative data and information on inaccessibility during 2010-2020 based on participants´ own experiences of inaccessibility in time and space: one questionnaire was solicited from each of the State Polio emergency operating center (EOC) members and one questionnaire from each of the LGA team members. Participants provided recalled information on the accessibility or inaccessibility (full or partial) of LGA headquarters, major cities, and other settlements on a yearly/quarterly basis during the period under review (2010-2020). Participants were experienced health professionals who had lived in the LGAs and were willing to provide the requested information. Although the initial assessment was conducted independently, the EOC and LGA data were consolidated in teams for cross-checking to minimize recall differences. Heatmaps using a system of color-coding was used to graphically represent the different extent of inaccessibility of settlements within LGAs over the years. Inaccessibility was defined as the inability of civilians like health workers and vaccination teams to safely move in and out of a given area due to the risk of attack by insurgents [18].

**Nutritional services and inaccessibility:** additional data to estimate the inaccessibility status of the LGAs were also obtained. The armed conflict in Borno and Yobe States led to increasingly severe food insecurity and undernutrition. Since 2010, UNICEF has supported the integration of community-based management of acute malnutrition (CMAM) and admission malnutrion treatment services (severe acute malnutrition (SAM) treatment) into 75% of fixed health facilities in North-eastern Nigeria [19]. Records of constraints in the movement of aid workers and security-compromised access at the community level to distribute food in different settlements within LGAs over time were used as a proxy to evaluate inaccessibility status [8,19]: settlements where food could be distributed easily were considered accessible, while those where food was impossible or difficult to be distributed due to the security situation were considered inaccessible.

**Satellite imagery population displacement data:** to determine the extent of reliability of inaccessibility based on health worker recall data of remaining populations, we analyzed the population displacement in Borno State based on satellite imagery estimates to assess the number of children aged <5 years residing in those populations over time. Satellite imagery estimation was obtained by using high-resolution commercial satellite imagery from DigitalGlobe´s WorldView-1, WorldView-2, WorldView-3, and GeoEye-1 satellites as previously described [5]. This imagery consisted of panchromatic and pan-sharpened color imagery at resolutions ranging from 31 to 50 cm [5,20] that was used to manually review each settlement in the conflict-affected areas of Borno State over multiple points in time. The settlement data were then grouped by LGAs. The assumption was that population displacement was synonymous with fleeing from violent, security-compromised inaccessible areas and was therefore taken as a measure of inaccessibility. Satellite imagery data were not available for Yobe State.

**Data analysis:** all sources of data on inaccessibility were aggregated at the LGA level. The extent of inaccessibility was based on an inassesibility threshold of 50%. Local government areas with >50-99% inaccessibility were described as “>50% inaccessible” and those with 100%, as “inaccessible”. Those LGAs with 1-49% inaccessibility were described as “>50% accessible” and those with 0% as “accessible”. Data were analyzed using Microsoft Excel on a Windows 2020 platform and tested by Pearson´s correlation.

**Ethics and approvals:** the study was reviewed by the Human Subjects Office and determined to be public health surveillance rather than research: it is a surveillance activity using recorded and recall data from health care and nutrition personnel or satellite imagery and did not involve collection of data or specimens from members of the public. This study was therefore cleared by the US Centers for Disease Control and Prevention as non-research.

## Results

**Figure 2:**
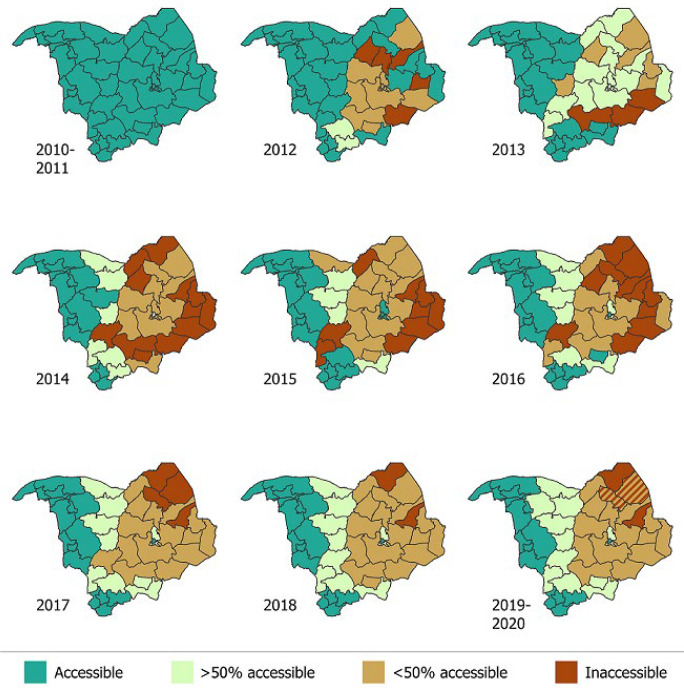
simplified Heatmaps of inaccessibility in Borno and Yobe States by Local Government Areas (LGA), 2010-2020

**Accessibility in local government areas of Borno and Yobe States:** the accessibility in LGAs of Borno and Yobe States on a yearly basis is depicted in Figure 2. These data indicate that during 2010-2011, all Borno and Yobe State LGAs were completely accessible (Figure 2). However, in 2012 while LGAs of Yobe State continued to be completely accessible, five LGAs (Dikwa, Gubio, Gwoza, Monguno and Nganzai LGAs) in Borno State were inaccessible. Late in the same year, nine LGAs in Borno had >50% inaccessibility. In 2013, Damaturu LGA in Yobe State started experiencing some >50% inaccessibility, but remained >50% accessible thereafter. In Borno, there was a gradual build-up in inaccessibility in Bama, Damboa and Gwoza. By 2014, there appeared to be general inaccessibility in LGAs in both states that continued until 2017 in affected Yobe LGAs and until 2020 in Borno state. The Borno LGAs of Bama, Chibok, Damboa, Dikwa, Gubio, Gwoza, Kala Balge, Mobbar, Ngala, Guzamala, Kukawa, Marte and Abadam and the Yobe LGAs of Gulani and Gubja experienced protracted periods of inaccessibility. Borno LGAs of Bayo, Biu, Kwaya Kusar, Shani, Hawul, and Maiduguri Metropolitan area and the remaining 11 LGAs of Yobe had remained completely accessible. The peak of inaccessibility was during 2014-2016 and varying thereafter.

**Figure 3:**
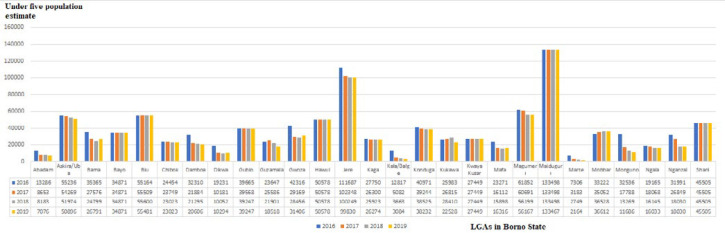
estimated populations of children aged <5 years in Borno State by Local Government Areas (LGAs), 2016-2019

**Figure 4:**
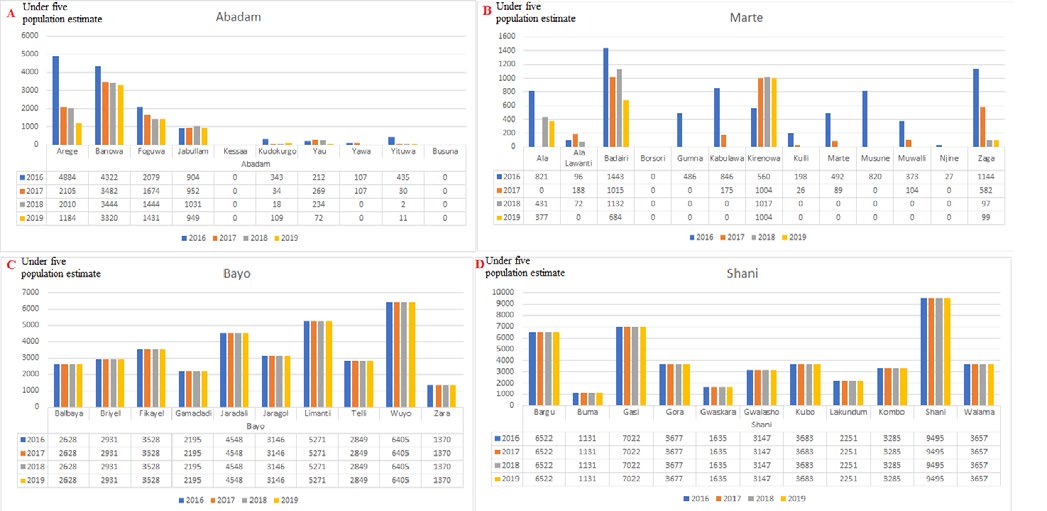
(A, B, C, D) estimated populations of children aged <5 years in Borno State by inaccessibility wards, 2016-2019

**Figure 5:**
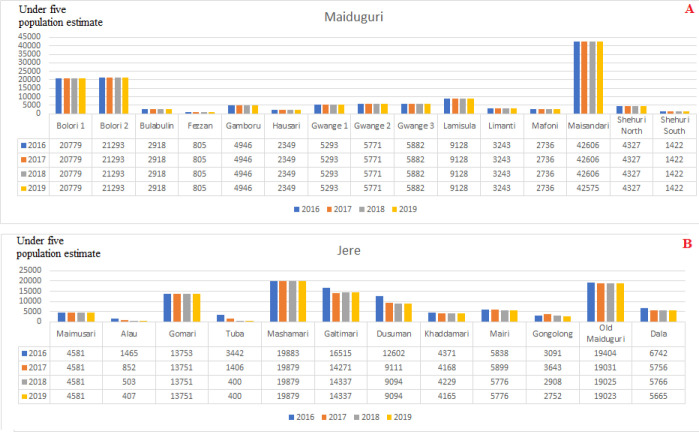
(A, B) estimated populations of children aged <5 years in Borno State in populated wards, 2016-2019

**Populations displacement based on satellite imagery data:** the estimated population of children aged <5 years in Borno State by LGA is shown in Figure 3. Satellite imagery data were available only for Borno State and for the period from 2016 to 2019. In all, 20 LGAs (74.1%) of Borno State experienced decreasing population in varying degrees during the period. Five LGAs - Abadam, Dikwa, Kala Balge, Marte and Monguno - experienced a >50% drop in the child population from 2016 to 2019. Mobbar LGA experienced slight increases in the child population from 2016 to 2019 (Figure 3). Examination of population displacement by ward in examples of inaccessible LGAs of Abadam and Marte (Figure 4), showed an irregular displacement pattern, with three of 10 (30%) wards in Abadam and 9 of 13 (69.2%) wards in Marte completely abandoned. Six LGAs-Bayo, Biu, Hawul, Kwaya Kusar, Maiduguri, Shani-had a stable child population. Investigation of population displacement in the two most populated Borno LGAs, Maiduguri and Jere, showed a stable population in all 16 wards in Metropolitan Maiduguri after 2016 (Figure 5). This also correlates with the accessibility of the LGA starting during 2015-2020 (Figure 2). Nine of 12 wards in Jere LGA experienced varying degrees of population displacement during 2016-2019. However, 2 wards in Jere experienced over 50% displacement (Figure 5).

**Nutritional services and insecurity in Borno and Yobe:** regarding nutritional service delivery, trends in SAM admissions and scale-up of CMAM service delivery units in Borno and Yobe States showed a steady increase during 2014-2016. Increased number of sites delivering services, and number of children admitted for SAM treatment, reflected the intensity of nutritional deficiency and need for treatment. During this time, humanitarian aid workers had to rely on local collaborators to deliver the much-needed services due to lack of or limited secure access. Constraints to entry by aid workers peaked during 2014-2017 were equated with security-compromised inaccessibility. The correlation between assessed inaccessibility by health workers and population displacement by satellite imagery comparison was determined for Borno State. The six LGAs with relatively stable populations were fully accessible, while those with a greater extent of population displacement showed increased inaccessibility. Bayo, Hawul, Kwaya Kusar and Shani LGAs experienced zero population displacement during 2016-2019 and remained accessible. On the other hand, eighteen LGAs - Marte, Abadam, Kukawa, Guzamala, Bama, Damboa, Dikwa, Gubio, Gwoza, Kaga, Kala/Balge, Konduga, Mafa, Magumeri, Mobbar, Mungono, Ngala, and Nganzai - with massive population displacement to other areas were >50% or completely inaccessible (Figure 2, Figure 3).

## Discussion

Prior to discovery of ongoing, previously undetected transmission of WPV1 there was evidence of the suboptimal seroprevalence to poliovirus in accessible areas, and a large pool of susceptible infants in both Borno and Yobe States [21]. The last reported cases of WPV in Nigeria and the entire WHO African Region occurred in four children liberated from trapped inaccessible locations and tested in IDP areas of Borno, Nigeria in 2016 [2]. Even though other WPV cases had been reported up through July 2014, genomic sequence analysis of those isolates showed the closest genetic linkage to isolates for cases reported in 2011 [22]. This indicates the critical disruptive role that prolonged insurgency and inaccessibility can play in allowing poliovirus transmission to escape prevention and detection for years. Our study aimed to determine the historical inaccessibility status of LGAs in Borno and Yobe States, Nigeria and provide data for mathematical modeling. This modeling provided an assessment of the penetration and the impact of innovative polio eradication initiatives on decreasing the likelihood of persistent WPV transmission, as well as the value and effectiveness of innovative surveillance activities in those areas [1].

Analysis of inaccessibility based on recalled information by field personnel and satellite imagery, population displacement data for 2016-2019 were highly consistent [5]. Despite some armed conflict, Borno and Yobe States had no accessibility issues during 2010-2011 and 2010-2012, respectively [5]. During 2012, Boko Haram began occupying villages and controlling large geographic areas in Borno including entire LGAs leading to population displacement; this began in 2013 in Yobe [5]. This suggests that insurgency did not play a major role in limiting polio elimination efforts in Nigeria before 2012. Widespread insecurity and inaccessibility peaked during 2014-2017 and changed variably in affected LGAs up to 2020. United Nations Children’s Fund (UNICEF) reports indicated that the nutritional status of children in Borno and Yobe States deteriorated rapidly in 2015, peaked in severity in 2017 and stabilized in 2018 with some exceptions in hard-to-reach areas (mainly in Damasak, Gubio, Kukawa and Northern Yobe) [19]. These nutrition program data correlate with our reconstruction of access from polio program field staff and provides external validation of the assessed accuracy of inaccessibility from the latter. The inability to safely access individual children in a large number of settlements in part or all of LGAs impacted the adequate provision of vaccination and surveillance services. Insurgency led to population displacement, increased vaccine-preventable disease transmission, malnutrition, poverty and psychological trauma [5,7,9]. These issues shaped the government´s responses to insurgency and perceptions of impact. Protracted inaccessibility in some LGAs provided for favorable conditions for silent WPV transmission to occur, as evidenced many years prior to 2016 in Borno State, Nigeria. Inaccessabillty is similarly implicated in the current persistent transmission and confinement of WPV1 in major portions of Pakistan and Afghanistan [2,14,15,23]. Active surveillance was limited to accessible portions of Borno and Yobe LGAs but was unlikely to be optimal given population displacement; in security-compromised areas, it was not feasible to conduct AFP surveillance as evident by undetected WPV transmission until discovery in 2016 [2,3,4,12,18].

During 2012-2019, settlements in many LGAs in Borno and Yobe States experienced armed conflict and insurgency control that jeopardized key components of the polio eradication efforts. At the time that continued endemic transmission of WPV was discovered, satellite imagery with habitation assessment and estimated settlement population in December 2016 indicated approximately 468,800 children aged <5 years were trapped in inaccessible areas of Borno State [2]; a subsequent assessment in December 2017 estimated 157,000 children aged <5 years who remained trapped in fully inaccessible areas [5]. Innovative strategies were used to vaccinate children remaining in many insecure areas, such as Reaching Every Settlement (RES), an initiative that allowed satellite-tracked vaccination teams to enter areas with limited security in conjunction with the armed civilian joint task force (cJTF), and Reaching Inaccessible Children (RIC) that engaged the military in entering insecure areas to vaccinate children while satellite-tracked and conduct spot surveillance. A surveillance initiative, Community Informants in Inaccessible Areas (CIIA) in which community members with cellphone access reported suspected AFP cases and guided caretakers to bring children to safe areas for investigation, stool specimen collection and subsequent testing, allowed for a restart of surveillance activities in these remaining insurgent-held areas [2,9,24]. In Borno State, the RIC initiative reached 123,561 children aged <5 years in 4,941 highly security-compromised, inaccessible communities with multiple doses of OPV during late-2017-mid-2020; the CIIA reached 5,691 inaccessible settlements, 1010 of which were previously unreached for surveillance by any other intervention (source: Borno polio EOC). Importantly, children in 886 (87.7%) of the 1010 previously unreached settlements were also reached with vaccination. With these program innovations continuing and liberation and escape of residents from many previously inaccessible settlements under occupation, only approximately 30,000 children aged <5 years remained unreached by polio vaccination in Borno in 2020 (source: Borno Polio EOC); small inaccessible populations remained in Bama, Dikwa, Gwoza, Ngala, Marte, Abadam, Kukawa, Guzamala LGAs in Borno State and Gujba and Gulani LGAs in Yobe. These initiatives coupled with engagement programs to combat vaccine skepticism and build trust for vaccine acceptance in areas that were always accessible led successfully to interruption of WPV transmission in Nigeria [1,25].

Limitations: our study comes with some limitations. The accuracy of retrospective recall data remains uncertain and may not provide a literal reproduction of the past. In our study, to minimize that potential recollection bias, we combined independent assessments from multiple individuals who were present in areas of inaccessibility and validated access with satellite imagery population displacement data. There were no satellite images prior to 2016, which prevents any corroboration of recalled information for 2012-2015.

## Conclusion

With the current transition of RIC and other polio intervention activities to State government personnel and the decreasing support of international partners, there is the need to sustain surveillance and childhood vaccination to preserve the area as poliovirus-free. Continuation of community-based poliovirus surveillance in inaccessible LGAs would be prudent, to the extent possible.

**Disclaimer:** the findings and conclusions in this report are those of the authors and do not necessarily represent the official position of the U.S. Centers for Disease Control and Prevention.

### What is known about this topic

- Areas with prolonged armed conflict are difficult for public health interventions such as polio vaccination and surveillance, thereby providing favorable conditions for possible undetected and sustained circulation of poliovirus or of other vaccine-preventable disease agents.

### What this study adds

- This study provides information on the extent of inaccessibility over time (2010-2020) and supports a very low probability of undetected wild poliovirus circulation within remaining trapped populations in security-compromised areas of Borno and Yobe States, Nigeria after 2016;
- Provides data on the dynamics of inaccessibility in Borno and Yobe Sates, Nigeria that is essential for program planning, management and for modelling vaccination and surveillance coverage in those areas to better understand the likelihood of undetected silent wild poliovirus circulation.
